# Trophic Ecology of the Tiger Shark (*Galeocerdo cuvier*) and Bengal Whipray (*Brevitrygon imbricata*) Harvested by Sri Lankan Fisheries Based on Stable Isotope Analysis

**DOI:** 10.1093/icb/icaf076

**Published:** 2025-07-10

**Authors:** Pathirannahalage Buddhi Maheshika Pathirana, Raven Harrison, Divia Feinstein, Jasmin Graham, Rose Leeger, Ashley Liao, Norah Mendoza, Lelah Munyer, Ashley D Mocorro Powell, Karson Burton-Reeder, Widanarachchige Sahan Thilakaratna, Sora Lee Kim

**Affiliations:** Ocean Rosy, Colombo 00600, Sri Lanka; Minorities in Shark Sciences, Sarasota, FL 34236, USA; Minorities in Shark Sciences, Sarasota, FL 34236, USA; Department of Life and Environmental Sciences, University of California, Merced, Merced CA 95343, USA; Minorities in Shark Sciences, Sarasota, FL 34236, USA; Department of Environmental Studies, University of Colorado Boulder, Boulder, CO 80309, USA; Department of Life and Environmental Sciences, University of California, Merced, Merced CA 95343, USA; Minorities in Shark Sciences, Sarasota, FL 34236, USA; Department of Life and Environmental Sciences, University of California, Merced, Merced CA 95343, USA; Minorities in Shark Sciences, Sarasota, FL 34236, USA; Department of Biological Sciences, University of the Pacific, Stockton, CA 95211, USA; Ocean Rosy, Colombo 00600, Sri Lanka; Department of Life and Environmental Sciences, University of California, Merced, Merced CA 95343, USA

## Abstract

Sri Lankan fisheries have substantial elasmobranch catches, but the local ecology of individual species is not well characterized. We examine the tiger shark (*Galeocerdo cuvier*) and Bengal whipray (*Brevitrygon imbricata*), two elasmobranch species with variable life history and feeding ecology that represent differing trophic guilds. Tiger sharks have a global distribution and are well-studied in some regions, but there is a lack of ecological information specific to the Indian Ocean. In contrast, Bengal whiprays are often misidentified at the species level and are thought to largely feed on benthic flatworms. Here, we investigate the trophic ecology of these two species with stable isotope analysis, which tracks the nutrient flow through food webs. Morphometric measurements and samples were obtained from tiger sharks [muscle (*n* = 24), teeth (*n* = 17)] and Bengal whiprays [muscle (*n* = 44)] after boats were onshore; tissues were sampled and dried before transport for stable isotope preparation. Both tiger sharks and Bengal whiprays have a wide range of δ^13^C values spanning from −17.8 to −14.8‰, indicating diverse feeding habitats. In general, tiger sharks have higher δ^15^N values (13.3 ± 0.6‰) than Bengal whiprays (12.1 ± 0.7‰), although five Bengal whiprays had similar δ^15^N values to tiger sharks. There were also δ^15^N differences by sex among Bengal whiprays, which suggests some foraging or baseline differences within the population. The isotopic differences among market locations were subtle and difficult to discern given differences in sample size. These insights into the ecology of tiger sharks and Bengal whiprays in Sri Lanka, along with other studies, including tagging and stomach content analysis, are critical in developing ecosystem-based management strategies. For example, the identification of essential habitats for the Marine Protected Area designation would restrict fishing and help mitigate impacts on population structure and dynamics, two critical considerations for these two species, which are listed as Near Threatened and Vulnerable, respectively, on the International Union for Conservation of Nature Red List.

## Introduction

Elasmobranchs (sharks, rays, and skates) play a crucial ecological role in coastal and open ocean regions (Heithaus et al. 2008; Baum and Worm 2009; Ferretti et al. 2010). Large predatory shark species undertake long-distance migrations (Estes et al. 2016) and shape the ecosystem at the community level directly via predation and indirectly via risk effects (Creel and Christianson 2008; Heithaus et al. 2008; Roff et al. 2016; Trystram et al. 2017). Hence, they promote species diversity, especially in the coral reef ecosystems, by controlling prey species and supporting reef health (Ruppert et al. 2013). Although the most well-studied elasmobranchs are apex predators, mesopredators play an important intermediate role between bottom-up and top-down processes in the marine food web (Ritchie and Johnson 2009; Navia et al. 2017). The foraging behaviors of mesopredatory rays create bioturbation, which enhances habitat heterogeneity and productivity for other demersal and juvenile fish species (Dulvy et al. 2014). Since apex predator sharks and mesopredator rays perform distinct ecological functions, their population declines create different changes in the marine food web (Heithaus et al. 2008). The decline of apex predator shark species is shown to have cascading effects with an increase in the abundance of mesopredators, such as smaller elasmobranchs, which serve as prey species (Stevens et al. 2000; Dulvy et al. 2004; Myers et al. 2007; Baum and Worm 2009; Ferretti et al. 2010). Elasmobranchs suffer population declines primarily due to overfishing, bycatch, pollution, and habitat degradation (Lucifora et al. 2011; Fortuna et al. 2024). Apex predator sharks regulate upper food web dynamics and stabilize ecosystems, while mesopredator rays influence benthic productivity and energy flow; therefore, Sri Lankan fisheries management needs to consider these species not merely as “stocks” but as functional ecosystem components that will help to maintain ecological integrity and secure long-term sustainable fisheries. However, limited data on the ecology of many elasmobranch species, especially from the Global South, including Sri Lanka, hinders the management efforts. Therefore, it is vital to elucidate the trophic ecology of species to trace energy flow within ecosystems, especially as it changes with sex and ontogeny, as ecological roles may vary with these biological traits (Navia et al. 2017). Conversely, many elasmobranch diet studies rely on stomach content analysis (SCA), but this technique requires a large sample size, and specimens often have empty stomachs (Wetherbee et al. 2004). If stomach contents are present, only recently consumed prey species are available (Cortes 1999; Christensen and Moore 2009), which may not accurately reveal the diet composition since soft parts are easily digested (Christensen and Moore 2009).

Recently, stable isotope analysis (SIA) emerged as a method to explore the diet and habitat preferences of elasmobranchs (Shiffman et al. 2012). The two most common stable isotope systems used in trophic ecology are carbon (^13^C/^12^C) and nitrogen (^15^N/^14^N), which are transferred from prey to consumer. Metabolic processes differentiate between isotopes so that ^12^C is preferentially respired in carbon dioxide and ^14^N is preferentially excreted in urea waste, while heavier isotopes are retained in protein substrates (e.g., muscle and collagen) of consumers. In general, carbon and nitrogen isotopes track energy flow through an ecosystem, and there are baseline differences that vary with productivity regimes (i.e., near vs. offshore). However, nitrogen isotopes differentiate more substantially with trophic levels and are often interpreted as a trophic indicator, especially for species with similar foraging habitats. SIA has elucidated individual and population-level ecological traits such as resource partitioning (Valls et al. 2017; Mulas et al. 2019), competition (Lea et al. 2019), and energy transfer (Mourier et al. 2016), as well as insights into macroecology (Ebert and Bizzarro 2009; Barbini et al. 2018), food web structure (Navia et al. 2017), and ecotrophic models (Coll et al. 2013).

One challenge to using SIA in food web studies for Sri Lanka is the lack of prior ecological or oceanographic studies to discern isotopic baseline gradients. Moreover, Sri Lankan field researchers currently lack access to research institutions conducting resource-intensive research, particularly related to the SIA of threatened marine species. This limitation hinders efforts to obtain a clear understanding of elasmobranch trophic ecology. There are tissue-specific trophic discrimination factors (TDF) and incorporation rates based on amino acid levels and metabolic activity. For example, the TDFs estimated for leopard sharks (*Triakis semifasciata*) muscle and dental collagen δ^13^C values are 1.7 ± 0.5‰ and 4.7 ± 0.5‰, respectively, while δ^15^N values are 3.7 ± 0.4‰ and 2.0 ± 0.7‰, respectively, which are due to the organic compound constituents in each substrate (Kim et al. 2012; Zeichner et al. 2017). After consideration of tissue-specific TDFs, the isotope composition can be compared between two tissues with different incorporation rates to reveal the stability or variation in diet through time. Muscle is often used for SIA, and the residence time (1/incorporation rate) estimated for leopard sharks is 214 and 325 days for carbon and nitrogen, respectively (Kim et al. 2012). Tooth collagen also provides another protein substrate that offers dietary “snapshots” in elasmobranchs since their teeth are formed below the epithelial surface, then move in a conveyor belt system to the functional position. In the experiment with leopard sharks used to estimate TDF and incorporation rate for muscle, the residence time for tooth collagen was 42–48 days with a 240–265 day delay from formation to functional position (Zeichner et al. 2017). A comparison of isotopic composition between tissues with varying incorporation rates allows an assessment of diet and habitat variation through time despite the lack of a prior information to provide additional context for SIA.

Tiger sharks (*Galeocerdo cuvier*) are globally distributed throughout tropical and subtropical habitats in coastal to pelagic waters (Randall 1992; Domingo et al. 2016). These large, apex predators can reach 550 cm in total length (TL) (Meyer et al. 2014; Dicken et al. 2016) and undergo long-distance migrations with differences among individuals (Holmes et al. 2014; Werry et al. 2014). From SCA and SIA, tiger sharks are known to feed on a diverse range of prey, including teleosts, elasmobranchs, crustaceans, molluscs, marine mammals, reptiles, and birds (Simpfendorfer 1992; Lowe et al. 1996; Simpfendorfer et al. 2001), and are thought to undergo an ontogenetic diet shift (Simpfendorfer 1992; Lowe et al. 1996). In addition, they are known to structure the environments they inhabit (Heithaus et al. 2012; Burkholder et al. 2013) by triggering “risk effects” on several prey, including green sea turtles (Heithaus et al. 2007) and dugongs (Wirsing et al. 2007). In Sri Lanka, tiger sharks are locally known as “Koti mora” and “Thalagoi mora” in Sinhalese and “Valluvan sorrah” and “Kurangu sorrah” in Tamil (De Bruin et al. 1994). Several local studies report the presence of tiger sharks (De Bruin et al. 1994; Herath 2012) mostly caught by longlines around Sri Lanka (Moron et al. 1998). Tiger sharks are the third most harvested shark species in offshore pelagic waters by Sri Lankan fishers (Ocean Rosy, unpublished data), but most elasmobranch studies originating in Sri Lanka focus on silky (*Carcharhinus falciformis*) and blue (*Prionace glauca*) sharks. The diet studies of tiger sharks focus on Australia (Heithaus 2001; Simpfendorfer et al. 2001), Hawaii (Lowe et al. 1996), and Atlantic (Aines et al. 2018) waters. However, to date, the habitat use, diet, and trophic ecology of tiger sharks within the exclusive economic zone around Sri Lanka and the greater Indian Ocean remain understudied.

*Brevitrygon imbricata* is commonly known as the Bengal whipray (Last 2016) but also referred to as the scaly whipray in recent literature (Last et al. 2023) and previously known as *Himantura imbricatus* (De Bruin et al. 1994; De Silva 2006) and *Himantura imbricata* (Moron et al. 1998). The Bengal whipray is a small benthic ray found along the inner continental shelves of the northern Indian Ocean, including the Bay of Bengal to further south in the Andaman Sea (Last 2016). This species inhabits the coastal waters of India, Bangladesh, Indonesia, Malaysia, Myanmar, and Thailand (Sherman et al. 2021), and several studies reveal the presence in the coastal waters of Sri Lanka (Last et al. 2023). The Bengal whipray is the second most harvested ray species in the coastal waters of Sri Lanka (Ocean Rosy, unpublished data) and is known to feed on bottom-living invertebrates (Rainboth 1996; Carpenter et al. 1997). Although presence is confirmed, no study has addressed the diet and trophic ecology of the Bengal whipray in the coastal waters of Sri Lanka.

Here, we characterize the trophic ecology of the infamous apex predator, tiger shark, and the less-studied mesopredator, Bengal whipray, caught in waters surrounding Sri Lanka. We use muscle δ^13^C and δ^15^N values to discern differences in diet and/or habitat preference between these two elasmobranch species with differing morphology and life history with respect to species, size, and sex. In addition, we compare muscle protein and dental collagen δ^13^C and δ^15^N values from tiger sharks for insights to dietary variation through time. Although we lack stable isotope data for potential prey and baseline gradients, we are able to compare patterns of trophic ecology with respect to sex and ontogeny between two species representing different ecological guilds—meso- and apex predators—caught by Sri Lankan fishers in coastal and international waters. These findings from SIA, along with future tagging and SCA efforts, could identify critical habitats and migration corridors for tiger sharks and Bengal whiprays in Sri Lankan waters, which is crucial for effective fisheries management and establishing Marine Protected Areas.

## Materials and methodology

### Specimen collection

**Fig. 1:**
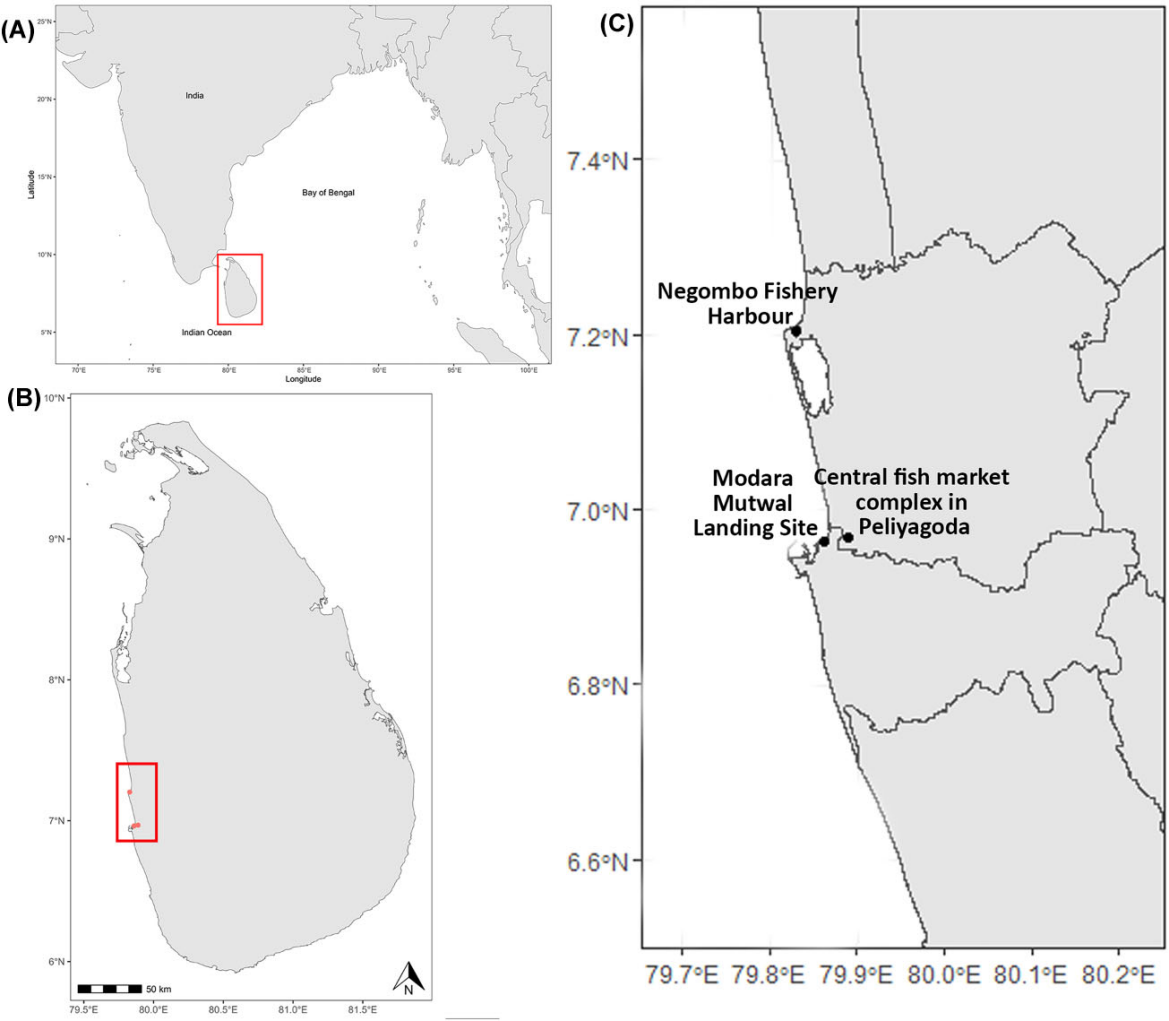
Map of sampling locations. (A) The location of Sri Lanka in the northern Indian Ocean and (B) the three markets in Sri Lanka where specimens were acquired: Negombo Fishery Harbour (NFH), the Central Fish Market Complex in Peliyagoda (PFM), and Modara Mutwal Landing Site and Fish Market (MFM), including (C) a close-up of the harbour/markets located on the western coast of Sri Lanka.

All specimens in this study were collected from fish markets in Sri Lanka from 27th June to 13th August 2024. Tiger shark samples were collected from Negombo Fishery Harbour (NFH) and included 24 muscle and 17 tooth specimens. Bengal whipray specimens were purchased from NFH, a Modara Mutwal Landing Site and Fish Market (MFM), and a Central Fish Market Complex in Peliyagoda (PFM) (Fig. 1). Bengal whipray specimens were processed for muscle sample collection (*n* = 44) at the Ocean Rosy Home Laboratory in Colombo, Sri Lanka. SCA was not possible given the lack of laboratory resources, and prey samples were not available since specimens were purchased from fish markets.

Biological metadata, including photos, were recorded for all specimens with all length measurements in cm. TL was obtained for each tiger shark specimen, and disc width (DW) for each Bengal whipray specimen. Additionally, inner clasper length and outer clasper length were measured in both species. The weights in kg were recorded only for Bengal whipray since many of the tiger sharks were gutted or cut in half before being loaded onto the measuring platforms available at the harbours. After collecting samples from specimens, tissues were transported to the lab on ice for up to 24 h, then frozen until preparation. All data, including biological and metadata, are available in the ICONIC Oceans Database (Pathirana 2025).

### Sample preparation

Muscle and tooth samples were dried at 50–60°C overnight to prevent decomposition and subsequently shipped to the University of California, Merced for SIA preparation. We dissected muscle samples (*N* = 68) to remove cartilaginous filaments, then ∼10 mg of tissue was transferred to a glass scintillation vial for lipid and urea extraction following methods of Kim and Koch (2012). The samples were immersed in ∼10 mL of petroleum ether, agitated in an ultrasonicator for 10 min, and then the supernatant was removed. This process was repeated two more times, leading to a total of three rinses in petroleum ether. For the urea extraction, 10–20 mL of DIW was added into a scintillation vial and ultrasonicated for 10 min. The DIW was then decanted into a waste beaker. These steps were repeated three times to completely remove urea from the tissue samples. After completing these treatments, the muscle samples were either dried in an oven set to 50°C or freeze-dried overnight. For the tiger shark tooth samples, enameloid was removed from the tooth samples utilizing a diamond-tipped bit on a Dremel. Although we collected 17 tooth samples, only 14 were prepared for collagen analysis. Longitudinal sections were cut from the teeth at a width of 0.75 mm. Tooth slivers were demineralized with 0.1M HCl for 1 h to isolate dental collagen (Trayler et al. 2023). Muscle protein or dental collagen samples with C:N > 4 were not considered in data analysis since δ^13^C values are likely affected by lipid content or incomplete demineralization (Kim and Koch 2012).

Dried samples for SIA were weighed into tin capsules measuring 3 × 5 mm (Costech). Samples were weighed in the range of 500 to 700 μg. Analysis was conducted at the Stable Isotope Ecosystem Laboratory of UC Merced (SIELO) utilizing a Costech 4010 Elemental Analyzer coupled with Conflo IV to a Delta V Plus Continuous Flow Isotope Ratio Mass Spectrometer. The δ^13^C and δ^15^N values were corrected for instrument drift, mass linearity, and standardized to international references of Vienna-PeeDee Belemnite for δ^13^C values and Atmospheric Nitrogen (AIR) for δ^15^N values, using certified reference materials USGS 41a and USGS 40 as well as an internal standard (Mb squid). Mean isotope compositions for reference materials were USGS 41a = 36.6 ± 0.2‰, USGS 40 = −26.4 ± 0.1‰ for δ^13^C values and USGS 41a = 47.6 ± 0.2‰ and USGS 40 = −4.56 ± 0.2‰ for δ^15^N values, which is within expected analytical error.

### Statistical analysis

To test if there was a significant difference in mean isotope values between species and sexes, we used one-way ANOVA or Kruskal–Wallis tests. If the results were normally distributed based on a Shapiro–Wilk test and met the assumption of variance homogeneity between groups based on a Fligner–Killeen test, we used the parametric ANOVA test. However, if these assumptions were not met, which is likely due to small sample size, we used the non-parametric Kruskal–Wallis test. In both cases, we evaluated the *P*-value with a significance level of α = 0.05. The name and test statistics are provided within the results with *P*-value. We also performed linear regressions for TL or DW to determine ontogenetic differences by species and sex using the “lm” function in R. The *r*^2^ and *P*-value are reported for all linear regressions, but the linear equation is only reported when the relationship is significant. Comparisons between individual muscle and teeth stable isotope values were based on a non-parametric Wilcoxon Signed-Rank test, given these were paired samples, but a small sample size. Additionally, to compare the isotopic niches of the species, we used SIBER (Stable Isotope Bayesian Ellipses in R) for estimating the standard ellipse and convex hull areas. The standard ellipse area (SEA) was corrected to minimize bias for small sample sizes using Bayesian Inference, following the methodology of Jackson et al. (2011). All analyses were performed in R version 4.4.3.

## Results

Our results include the muscle samples (*n* = 24) and tooth samples (*n* = 14) of tiger shark and muscle samples (*n* = 44) of Bengal whipray (Table 1), additional biological metadata are available in the ICONIC Oceans Database (Pathirana 2025).

**Table 1:** Summary of stable isotope values (δ^13^C, δ^15^N, and C:N) in muscle and tooth samples of tiger shark and Bengal whipray.

Species	*N*	Tissue	δ^13^C	δ^15^N	C:N
			Mean ± SD	Mean ± SD	Mean ± SD
Tiger shark	24	Muscle	−16.6 ± 0.7	13.3 ± 0.6	3.3 ± 0.1
	14	Teeth	−13.9 ± 1.7	11.5 ± 0.6	3.0 ± 0.2
Bengal whipray	44	Muscle	−16.1 ± 0.6	12.1 ± 0.7	3.3 ± 0.2

**Fig. 2:**
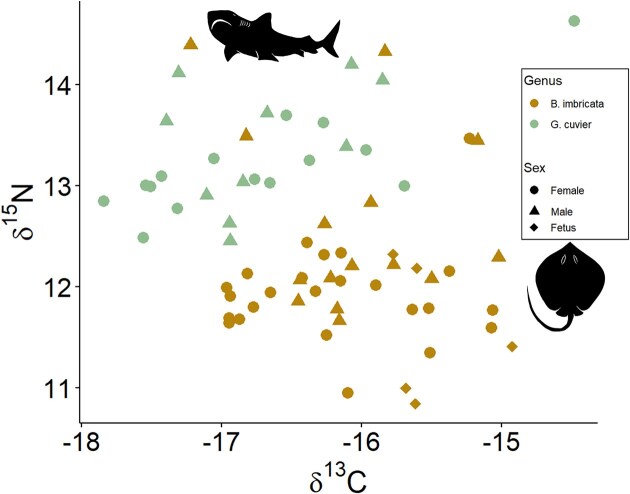
A comparison between common meso- and apex predators, Bengal whipray and tiger shark, caught by Sri Lankan fishers. The muscle δ^13^C and δ^15^N values serve as indicators for trophic ecology and habitat use. Symbols denote sex, with circles (●) for females, triangles (▲) for males, and diamonds (♦) for fetuses. The SEA_C_ comparison for species is available in Fig. S1.

**Fig. 3:**
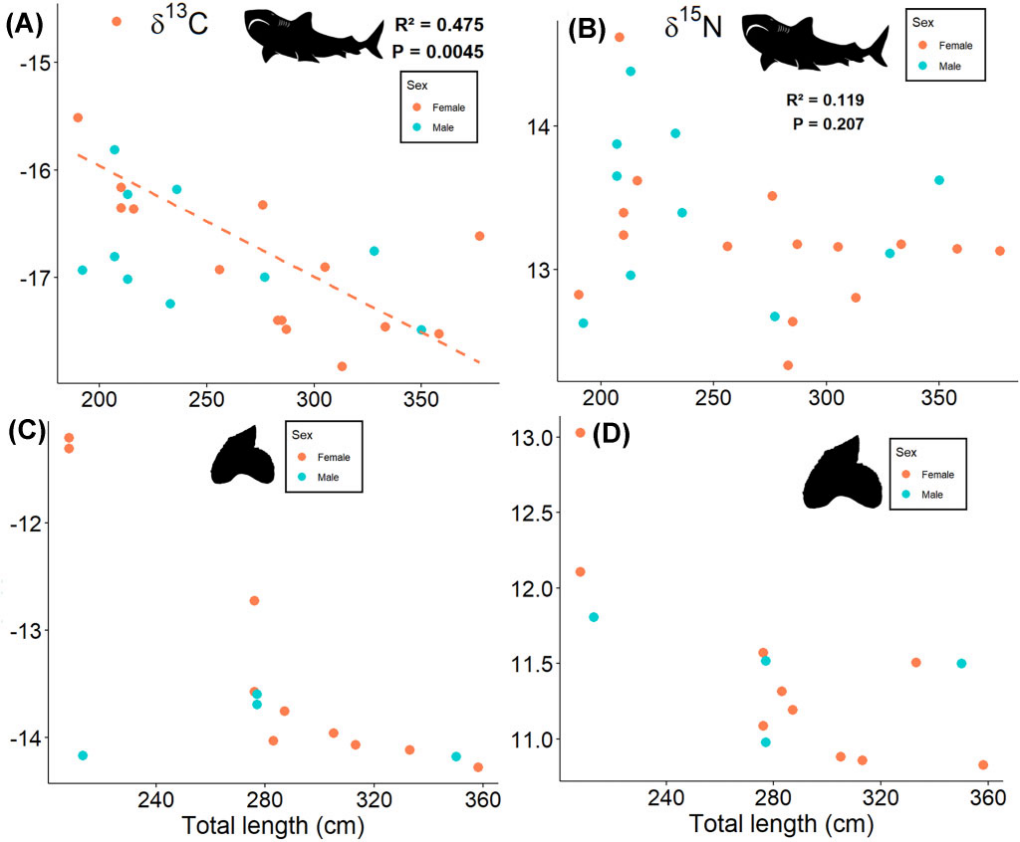
Elasmobranchs that are apex predators often exhibit ontogenetic changes in diet. A comparison of tiger shark total length (TL in cm) with (A) δ^13^C_Muscle_ and (B) δ^15^N_Muscle_ as well as (C) δ^13^C_Teeth_ and (D) δ^15^N_Teeth_ values reveals a significant correlation only for δ^13^C_Muscle_ values (δ^13^C_Muscle_ = −0.01 (TL) + −13.9, *r*^2^ = 0.475, *P* = 0.0045). The SEA_C_ for male and female tiger sharks is available in Fig. S3A.

**Fig. 4:**
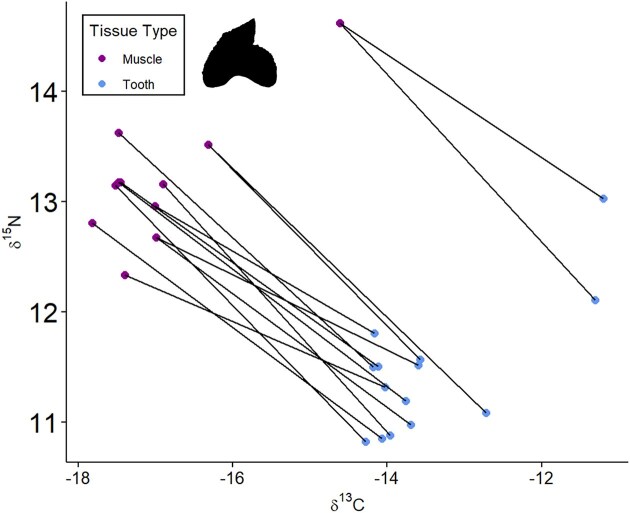
Muscle protein and dental collagen have differing amino acid compositions that produce variable isotopic offsets with diet as well as differing incorporation rates of dietary nutrients. A comparison between the stable isotope composition of these tissues from tiger sharks reveals significant differences between δ^13^C and δ^15^N as well as a consistent offset.

We analyzed the stable isotope composition of muscle protein and dental collagen for tiger sharks caught offshore from Sri Lanka. The TL of tiger sharks ranged 190–377 cm with a δ^13^C_Muscle_ range of −17.8 to −14.6‰ and a δ^15^N_Muscle_ range of 12.3 to 14.6‰ (Fig. 2). There was no significant difference between male and female tiger sharks for δ^13^C_Muscle_ values (one-way ANOVA; *F* = 0.004, *P* = 0.952) or δ^15^N_Muscle_ values (one-way ANOVA; *F* = 1.143, *P* = 0.297). A linear regression of tiger shark TL with isotope composition resulted in no correlation for δ^15^N_Muscle_ values (*r*^2^ = 0.119, *P* = 0.207), whereas there was significance for δ^13^C_Muscle_ values (δ^13^C_Muscle_ = −0.01(TL) + −13.9, *r*^2^ = 0.475, *P* = 0.0045) (Fig. 3A, B). Teeth were analyzed from a subset of tiger sharks (total 11 individuals, with upper and lower duplicates for 3 individuals, total tooth samples *n* = 14). The δ^13^C_Teeth_ values ranged from −14.2 to −11.2‰, and δ^15^N_Teeth_ values ranged from 10.8 to 13.0‰, which had no significant differences with sex (δ^13^C_Teeth_ Kruskal–Wallis test: χ² = 0.72, df = 1, *P* = 0.396 and δ^15^N_Teeth_ Kruskal-Wallis test: χ² = 0.32, df = 1, *P* = 0.572; Figs. 3C, D). There was a significant difference between muscle and tooth δ^13^C values (Wilcoxon Signed-Rank Test; *V* = 151, *P* = 4.6 × 10^-5^) and δ^15^N values (Wilcoxon Signed-Rank Test; *V* = 0, *P* = 1.5 × 10^-5^) with a consistent offset: δ^13^C_Teeth_—δ^13^C_Muscle_ = 3.3 ± 0.3‰ and δ^15^N_Teeth_—δ^15^N_Muscle_ = −1.8 ± 0.5‰ (Fig. 4). For three individuals, we collected upper and lower teeth, within individual differences ranged from 0.1 to 0.9‰. The mean C:N for muscle was 3.3 ± 0.1 and dental collagen 3.0 ± 0.2 (Table 1).

**Fig. 5:**
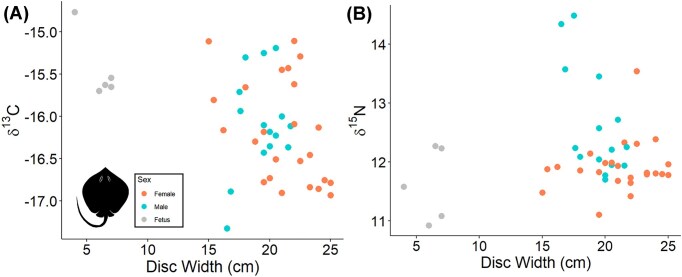
The Bengal whipray is a representative mesopredator in marine habitats of Sri Lanka. A comparison of fetus, female, and male DW to (A) δ^13^C and (B) δ^15^N values indicates minimal differences with ontogeny and sex.

The DW of Bengal whipray ranged from 15 to 25 cm with δ^13^C_Muscle_ range of −17.3 to −14.8‰ and δ^15^N_Muscle_ range of 10.9 to 14.5‰ (Fig. 2). The mean C:N for muscle was 3.3 ± 0.2. (Table 1). There were no significant difference in δ¹³C_Muscle_ values between males and females (Kruskal–Wallis test: χ² = 3.36, df = 2, *P*-value = 0.186). However, δ¹⁵N_Muscle_ values were significantly higher in males than females (Kruskal–Wallis test: χ² = 12.75, df = 2, *P*-value = 0.001). We compared between market locations for the Bengal whipray, but there were minimal differences in stable isotope composition and uneven sampling, which prevented robust statistical comparisons. A linear regression of DW to δ^15^N_Muscle_ values indicated no significant correlations for females δ^15^N_Muscle_ (*r*^2^ = 0.038 and *P* = 0.359) while a significant correlation was observed for female δ^13^C_Muscle_ values (r² = 0.174, P = 0.043). For males, DW was significantly correlated with δ^15^N_Muscle_ (*r*^2^ = 0.399 and *P* = 0.015), but no significant correlation was found with δ^13^C_Muscle_ (*r*^2^ = 0.119 and *P* = 0.227) (Fig. 5).

Tiger sharks generally exhibit higher δ^15^N values, indicating a higher trophic level, while Bengal whiprays have lower δ^15^N values, suggesting differences in trophic ecology (Fig. 2). To characterize the isotopic niche of these species, we compared 40% corrected standard ellipse areas (SEA_c_) with SIBER (Fig. S1). The mean SEA_c_ for all tiger sharks is 1.10 (Fig. S2), with similar SEA_C_ for females (1.13) and males (1.00) (Fig. S3A). In contrast, Bengal whiprays have a higher population mean SEA_C_ = 1.46 (including fetuses) (Fig. S2) with differences between males (1.76) and females (0.88) (Fig. S3B). The Bengal whipray SEA_C_ are impacted by the five individuals with high δ^15^N values, but based on the C:N values and QAQC metrics, these data are accurate and reflect the population variability. Overall, the stable isotope results indicate differences in isotopic niche between the tiger shark and Bengal whipray, representative species for an apex and meso-predator.

## Discussion

Our results indicate dietary overlap between the Bengal whipray, a demersal mesopredator, and the tiger shark, a generalist apex predator. Ontogenetic or sex-based differences were observed in Bengal whiprays for female as indicated by δ^13^C_Muscle_ values and male δ¹⁵N_Muscle_ values. Additionally, sex-based differences in isotopic niche were evident based on SEA_C_ analysis. Further, five individuals had elevated δ^15^N values, which impacted the isotopic niche of the population and male Bengal whiprays. In contrast, tiger shark δ^13^C_Muscle_ values had an inverse relationship with TL but not δ^15^N_Muscle_ values, indicating potential use of different habitats but similar trophic level prey through ontogeny. There was no indication of sexual segregation among tiger sharks based on statistical tests or SEA_C_. Finally, there was a consistent offset between tiger shark muscle protein and dental collagen stable isotope compositions that match offsets from a captive feeding experiment (Kim et al. 2012; Zeichner et al. 2017), suggesting relatively stable diets over 1 year. We recognize the limitations of our dataset without prey δ^13^C and δ^15^N values, but hope this study spurs additional projects to elucidate the diet and trophic ecology of elasmobranchs and contribute to conservation and management efforts.

Detailed diet and trophic ecology studies specific to elasmobranchs caught in coastal Sri Lankan waters are scarce. There are only a few abstracts for small-scale studies on SCA of sharks and rays in Sri Lanka, and comprehensive research is still lacking, especially for elasmobranch species. For large sharks in particular, conducting SCA presents several challenges. One major obstacle is that fishers often remove the stomachs of the catch before landing to preserve the freshness of the meat, making it difficult for researchers to obtain complete specimens. Additionally, many of the specimens available at landing sites are in poor condition—some fishing vessels stay at sea for over a month, by which time most stomachs are either empty or the contents are fully digested and unidentifiable. To conduct SCA, whole specimens must be purchased, or researchers must own or charter their own boat, which is cost-prohibitive. These logistical limitations, along with a lack of sustained funding and access to freshly landed specimens, hinder the feasibility of consistent and reliable SCA work in the country.

Without SCA results, it is difficult to know what prey species to target for SIA. A lack of stable isotope values for prey limits dietary interpretation of stable isotope results, but comparisons of sex and ontogeny can reveal variation in trophic ecology and habitat use. In this study, we chose two species from differing ecological guilds that represent meso- and apex predators in Sri Lankan marine waters. We also acknowledge that specific diet interpretations are limited even with SCA and prey stable isotope values, given the extended incorporation rate for muscle tissue (∼1 year) coupled with seasonal or ontogenetic migration for many larger elasmobranchs. Below, we provide additional ecological context for the stable isotope results in this study.

### Ecology of *B. imbricata*

This study offers the first assessment of Bengal whipray (*B. imbricata*) foraging ecology with SIA and focuses on specimens from the coastal waters of Sri Lanka. This demersal whipray is found on the inner continental shelf (Last 2016) at depths of 3–55 m (Weigmann 2016). This species is often misidentified as other species in the group due to morphological similarities, ontogenetic variability, nomenclatural difficulty, and taxonomic disputes. Although Bengal whiprays are known to occur in the western Indian Ocean, their presence in southern India was only recently confirmed, including the coastal waters of Sri Lanka in the Chilaw, Lunawa, Negombo, and Talaimannar regions (Last et al. 2023). In Sri Lanka, Bengal whiprays are caught as bycatch in bottom set gillnets, bottom longlines, and shrimp trawl nets in the coastal waters (Ocean Rosy, unpublished data). Bengal whiprays have a maximum DW of 29 cm with both males and females reaching maturity at 21 cm DW (Kizhakudan et al. 2010). The specimens in our study range have a DW of 4–25 cm, and include fetuses with a DW of 4–7 cm. Many elasmobranch species are known to have sexual segregation (Clarke et al. 2014; Bowers 2024) and ontogenetic shifts in diet (Lowe et al. 1996; Barbini and Lucifora 2012; Kim et al. 2012) and/or habitat (Grubbs 2010; Chin et al. 2013). As a bottom-dwelling ray species, Bengal whiprays primarily feed on benthic invertebrates (Rainboth 1996; Carpenter et al. 1997). However, ontogenetic diet studies are not available for this species.

Rays are often thought to consume benthic, invertebrate prey, but previous studies reveal that larger rays can also feed on relatively larger bony fish (Collins et al. 2007; Marshall et al. 2008; Brown et al. 2012; Jacobsen and Bennett 2012). Though the specific diet of Bengal whiprays remains unknown, a subset of males with DW ranging from 16.5 to 21.7 cm and one female with DW = 17.5 cm have the highest δ^15^N_Muscle_ values (Fig. 5B), similar to tiger sharks (Fig. 2). Explanations for these high δ^15^N values are the consumption of prey with high trophic levels or baseline δ^15^N values. If these individuals were caught in nearshore areas with increased human waste, there could be elevated baseline δ^15^N values (e.g., Risk and Erdmann 2000; Wong et al. 2022). These four males and one female are an anomaly that cannot be explained by ontogeny, sex, or the fish market. There is lower isotopic variation for the largest female Bengal whiprays (DW > 22 cm; Fig. 5), which could be due to biochemical differences, spatial segregation, or dietary differences as suggested for other elasmobranchs (Delpiani et al. 2013). Some of the smallest Bengal whipray individuals are fetuses, which have relatively high δ^13^C values and similar δ^15^N values to larger individuals (Fig. 5), that likely reflects maternal nutrient transfer during gestation (Olin et al. 2011; Tamburin et al. 2019). Since the fetus isotope composition is not indicative of foraging or trophic ecology, we do not include these individuals in our statistical models. Further, the incorporation rate of dietary nutrients in elasmobranch muscle is considered to be ∼1 year, based on previous captive feeding studies (MacNeil et al. 2006; Kim et al. 2012). Hence, the isotopic composition for young of the year does not entirely reflect their diet and habitat. Although we lack potential prey stable isotope compositions to compare with the Bengal whipray, our results suggest minimal dietary differences with sex or ontogeny.

Overall, the δ^13^C variation among Bengal whipray specimens is low, which suggests minimal baseline differences in foraging habitat. To date, many studies find evidence of resource partitioning and niche variation among skate and ray species within a habitat, but ontogenetic or sex differences within a species are more subtle (Elston et al. 2020). Previous studies on various ray species highlighted that trophic level increased with body size (Vaudo and Heithaus 2011; Yamaguchi et al. 2012; O'Shea et al. 2013; Tilley and Strindberg 2013; Navia et al. 2017; Hara et al. 2018), though this is not true for all species (Rastgoo and Navarro 2017; Rastgoo et al. 2018; D'Alberto et al. 2022). The trophic level described for many ray species ranges from 3.1 to 4.5, indicating their role as secondary or tertiary level consumers (Flowers et al. 2021). In addition, the trophic level for *Brevitrygon walga* in the Gulf and Oman Sea was estimated to be 3.6 (Rastgoo and Navarro 2017) and 3.5 (Rastgoo et al. 2018). Although we lack isotopic results from prey, we hypothesize that Bengal whiprays are mesopredators with similar trophic levels to other *Brevitrygon* species. Finally, Bengal whiprays likely contribute substantially to the benthic community structure of coastal waters in Sri Lanka, especially in the Modara and Negombo regions.

### Ecology of *G. cuvier*

In contrast to Bengal whipray, tiger sharks demonstrate highly migratory behavior (Holmes et al. 2014; Werry et al. 2014; Lea et al. 2019) and frequent regions of high prey biomass (Lea et al. 2019). Although tiger sharks may migrate throughout the Indian Ocean, the δ^13^C variations of most individuals sampled in this study are fairly constrained and similar to the Bengal whipray (Fig. 2). The tiger shark δ^13^C_Muscle_ range in this study was −17.8 to  −14.6‰ (Fig. 3), compared to specimens from Australia that ranged from −15.1 to −8.6‰ (Heithaus et al. 2013). The δ^13^C range for various studies featuring tiger sharks differs, which reflects the baseline differences between regions for this cosmopolitan, migratory species (Salinas-de-León et al. 2019; Peterson et al. 2020). Although the movement of tiger sharks has not been tracked and baseline δ^13^C values for the Indian Ocean are not fully constrained, there are seasonal and latitudinal changes as evidenced by temperature and productivity (Vinayachandran et al. 2021). In addition to a range of habitats, tiger sharks are known to feed on a wide range of prey species, including teleosts, elasmobranchs, crustaceans, molluscs, marine mammals, reptiles, and birds (Simpfendorfer 1992; Lowe et al. 1996; Simpfendorfer et al. 2001). The mean δ^15^N_Muscle_ value recorded in this current study (13.3 ± 0.6‰) (Table 1) is similar to prior studies in the Galapagos marine reserve of Isabela Island (13.7 ± 0.7‰), Santa Cruz Island (13.4 ± 0.7‰) (Salinas-de-León et al. 2019), Florida Big Bend in the eastern Gulf of Mexico (13.1 ± 0.3‰) (Peterson et al. 2020), and eastern Australia (14.2 ± 1.0‰) (Lipscombe et al. 2024). We found no isotopic differences between male and female tiger sharks (Fig. 2), which corroborates previous studies (Galván-Magaña et al. 2019). Although there is evidence for ontogenetic dietary changes in tiger sharks (Simpfendorfer 1992; Lowe et al. 1996; Galván-Magaña et al. 2019), our δ^15^N results do not indicate a trophic shift despite our sampled size range (190–377 cm) encompassing the transition from juvenile to adult TL ∼230 cm (Lowe et al. 1996; Simpfendorfer et al. 2001) (Fig. 3B, D). The correlation between TL and δ^13^C values suggests differences in habitat use within the Indian Ocean with ontogeny (Fig. 3A, C). For tiger sharks, the δ^13^C variation reflects the range of foraging habitats, and the consistent δ^15^N values across populations could indicate its generalist diet.

While muscle integrates diet over the span of approximately 1 year, we also analyzed dental collagen, which provides a shorter snapshot of prior diet. Like most sharks, tiger sharks have a conveyor-belt system of tooth replacement where the functional tooth forms below the epithelial tissue, then moves from the lingual to the buccal position. The only quantitative estimate of incorporation rate into dental collagen and timing from formation to functional position is from a captive feeding study of leopard sharks (*T. semifasciata*) that indicates a 42–83 day incorporation rate, similar to plasma, and ∼250 day time lag (Zeichner et al. 2017). Based on this study, the expected difference between dental collagen to muscle (with propagated error) is 3.0 ± 0.7‰ and −1.7 ± 0.7‰ for carbon and nitrogen isotope composition, respectively (Zeichner et al. 2017). The δ^13^C_Teeth_ values of tiger shark specimens ranged from –14.2 to −11.2‰ and δ^15^N_Teeth_ values ranged from 10.8 to 13.0‰ and closely tracked the isotope composition of muscle (Fig. 4). The average dental collagen to muscle protein δ^13^C offset was 3.3 ± 0.3‰ and δ^15^N offset was −1.8 ± 0.5‰, mirroring the results of the captive *T. semifasciata* (Zeichner et al. 2017), which suggests the short term diet from 200 + days prior (e.g., stable isotope values of teeth) mirrors the longer integration time of diet over ∼1 year (e.g., stable isotope values of muscle). There was one individual (BM709), which had an anomalous relationship between δ^13^C_Teeth_ and δ^13^C_muscle_, but the dental collagen C:N > 4, suggesting incomplete demineralization. For some individuals, we analyzed the upper and lower teeth, and while there is some offset, the differences are minor, except for one female with the highest δ^13^C and δ^15^N values (Fig. 4). This difference between upper and lower teeth suggests there may be some seasonal differences in diet, but given that dental collagen and muscle δ^13^C and δ^15^N offsets align with expectations from prior studies, the diet of tiger sharks in waters surrounding Sri Lanka is relatively stable despite the highly seasonal upwelling and productivity in the northern Indian Ocean (Shankar et al. 2002; Vinayachandran et al. 2021).

### Interspecific differences among elasmobranchs

This study presents the first comparison of stable isotope ecology for two elasmobranch species representing meso- and apex predators in Sri Lankan coastal waters. Our results reveal distinct differences between the trophic ecology of Bengal whipray and tiger shark, following an expected pattern for trophic level as well as new insights into movement and baseline δ^13^C values. A relatively small ray, the Bengal whipray is a mesopredator with lower δ^15^N_Muscle_ values (12.1 ± 0.7‰) than tiger sharks (13.3 ± 0.6‰), an apex predator, but these species have similar and overlapping isotopic niche widths based on SEA_C_ (Fig. S1). Tiger sharks consume a larger proportion of fish (Vaudo and Heithaus 2011), have higher energetic needs for their pelagic lifestyle, and are an order of magnitude larger than Bengal whiprays. In contrast, Bengal whiprays feed on benthic, lower trophic level prey that likely have lower δ^15^N values (Abdullah et al. 2023), although foraging in coastal waters with nutrient subsidies from human water could elevate baseline δ^15^N values (e.g., Risk and Erdmann 2000; Wong et al. 2022).

Although tiger sharks and Bengal whiprays vary in their relative trophic levels based on δ^15^N_Muscle_ values, the δ^13^C_Muscle_ overlap between tiger sharks (−17.8 to −14.6‰) and Bengal whiprays (−17.3 to −14.8‰) suggests foraging within food webs with similar primary productivity sources (Fig. 2, Fig. S1). There is evidence of tiger sharks feeding primarily on coastal species, including rays, in the western Atlantic along the US continental shelf (Edman 2016). According to anecdotal data from artisanal fishers in the southern part of Sri Lanka, juvenile tiger sharks (*∼*100 cm in TL) inhabit coastal waters. Therefore, juvenile tiger sharks may share resources with Bengal whiprays. Another possible explanation for this overlap in δ^13^C_Muscle_ values could be that the muscle integrates isotopic values throughout tiger shark movement across open ocean, coastal lagoon, and reef ledges (Papastamatiou et al. 2009) and feeding from pelagic to benthic environments (Nakamura et al. 2011), which likely have variable baseline δ^13^C values. The consistent offset between muscle and teeth isotope composition in a subset of the sampled tiger sharks suggests relatively similar foraging patterns through time or minimal baseline differences (Fig. 4). Although tiger sharks are considered an apex predator, these results suggest a generalist diet that includes prey from benthic environments and consumption of low trophic level prey, similar to Bengal whiprays, which suggests an important and dynamic role linking an ecosystem’s food web.

The trophic differences between tiger sharks and Bengal whiprays are surprising due to their differences in mouth morphology, body size, and migration behavior. Many ray species partition resources based on body size (Smale and Cowley 1992), habitat use, mouth morphology, and movement behavior (Barbini and Lucifora 2012). We do note the five Bengal whipray individuals (4 males, 1 female) with higher δ^15^N_Muscle_ values, which could indicate a high trophic diet due to lack of competition, differing dental morphology, or unusual foraging behavior as highlighted for other ray species (Marshall et al. 2008; Vaudo and Heithaus 2011). However, comprehensive information on dentition is lacking for this species; in contrast to the tooth plates of durophagous rays, Bengal whiprays have small, weak teeth like other dasyatids (Kolmann et al. 2015; Lim et al. 2019). We also note that intra-specific dietary differences between juvenile and adult Bengal whiprays were not revealed via SIA, given the long incorporation rate of muscle. We suggest future SCA to provide finer and higher resolution results of diet composition (MacNeil et al. 2006; Elston et al. 2020). In addition, more specific interpretations of stable isotope results require sediment and/or prey sampling to discern baseline and prey differences, respectively, among sites and species. Overall, Bengal whipray is a poorly studied coastal ray species (Rainboth 1996; Carpenter et al. 1997), and further studies are needed to determine its diet in higher resolution beyond bottom-living invertebrates.

Though tiger sharks and Bengal whiprays are listed as “Near Threatened” and “Vulnerable” in the International Union for Conservation of Nature Red List, respectively, neither species is protected in Sri Lanka. Tiger sharks are susceptible to offshore drift longlines, while Bengal whiprays are harvested by coastal bottom-set gillnet, bottom longline, and shrimp trawlers. Hence, it is important to identify and protect the feeding grounds of these two species, and other sharks and rays in the coastal waters and the greater Indian Ocean around Sri Lanka via ecosystem-based management approaches and the declaration of Marine Protected Areas to restrict fisheries to preserve these species.

## Conclusion

These findings provide insight into the ecological role and resource use of two elasmobranch species with distinct life histories. Both Bengal whipray and tiger shark are highly understudied, and this project marks the first findings of its kind with stable isotope results from elasmobranchs in Sri Lankan ecosystems. Future directions include analysis of other organisms to better understand the food web dynamics of coastal Sri Lanka, as well as tagging efforts to determine more accurate movement patterns of tiger sharks and Bengal whiprays. In addition, stable isotope data could be complemented with other chemical techniques, such as trace metal or fatty acid composition, to better distinguish dietary differences. We hope this study spurs additional studies for tiger sharks and Bengal whiprays, and other understudied species, as well as initiates conservation efforts for elasmobranchs in Sri Lanka. This region is highly understudied for fisheries management and marine conservation; the MISS ICONIC Oceans program offers an opportunity for local scientists to lead data generation and expand their professional network. With the growing threat of climate change and human impacts on coastal fisheries and ecosystems, it is important to understand the potential effects on meso- and apex predators. The collaboration between MISS, Ocean Rosy, and ICONIC Oceans will enhance research, management, and advocacy efforts by integrating resources and expertise. This collaboration will result in more effective, inclusive, and impactful outcomes in promoting sustainable marine practices and protecting ecosystems, especially in data deficient regions, such as Sri Lanka.

## Author contributions

P. Buddhi Maheshika Pathirana: contributed to conceptualization, sample collection, data curation, formal analysis, writing – original draft preparation, writing review, and editing, and served as first and corresponding author. Sora Lee Kim: contributed to supervision, project administration, resources, writing – original draft preparation, writing review and editing, and served as corresponding author. Ashley Liao: contributed to data curation, formal analysis, Investigation, writing review, and editing. W. Sahan Thilakaratna: contributed to sample collection, writing – Original Draft Preparation, writing review, and editing. Raven Harrison: contributed to data curation, formal analysis, visualization, and writing original draft preparation. Divia Feinstein, Ashley D. Mocorro Powell, Rose Leeger, Karson Burton-Reeder, and Lelah Munyer: contributed to data curation, investigation, writing review, and editing. Norah Mendoza: investigation, contributed to project administration and resources. Jasmin Graham: contributed to conceptualization and funding acquisition.

## Supplementary Material

icaf076_Supplemental_File

## Data Availability

Biological metadata are available in the ICONIC Oceans Database (Pathirana, Buddhi. Stable Isotope Analysis of *Galeocerdo cuvier* and *Brevitrygon imbricata* in Sri Lanka. Minorities in Shark Sciences. (https://iconicoceans.misselasmo.org/)
