# Supplementary material for: Trophic Ecology of the Tiger Shark (Galeocerdo cuvier) and Bengal Whipray (Brevitrygon imbricata) Harvested by Sri Lankan Fisheries Based on Stable Isotope Analysis
Source: Integr Comp Biol. 2025 Jul 10;65(5):1280–93. doi: 10.1093/icb/icaf076 (PMC12596733; doi:10.1093/icb/icaf076)
Supplement: icaf076_Supplemental_File [file icaf076_supplemental_file.pdf]

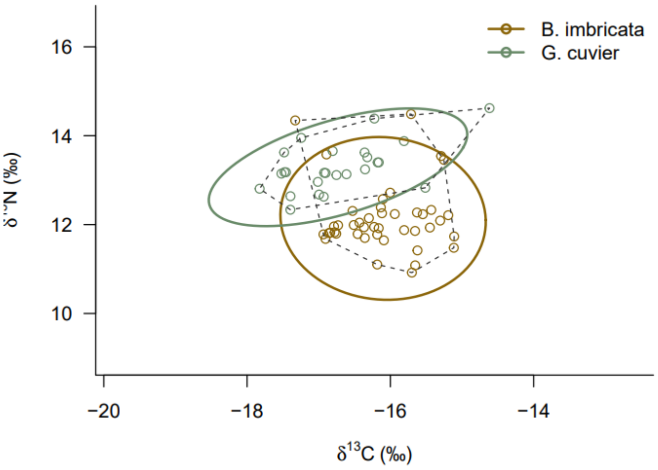

Figure S1: The standard ellipse area for tiger shark *Galeocerdo cuvier* and Bengal whipray *Brevitrygon imbricata* with corrected standard ellipse area (tolerance at 40%; solid lines) and convex hull area (dashed lines). See main text for metrics.

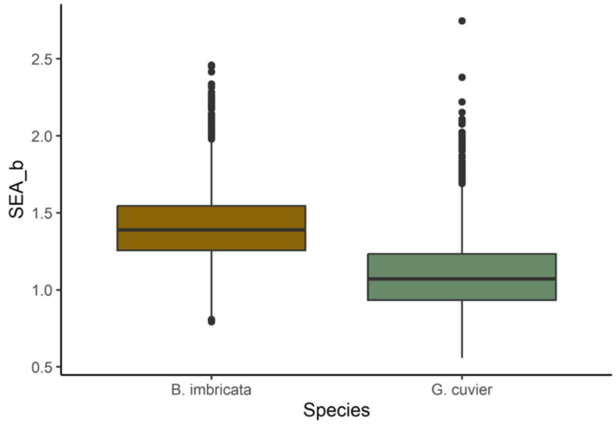

Figure S2: The distribution of SEA_c differ between tiger shark *Galeocerdo cuvier* and Bengal whipray *Brevitrygon imbricata* indicating differences in isotopic niche width.

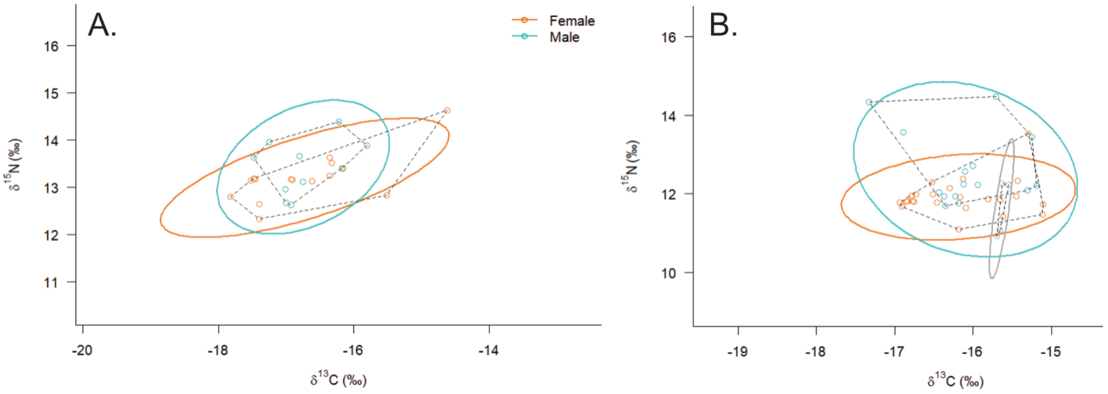

Figure S3: The SEA_c differentiation for (A) tiger sharks and (B) Bengal whiprays between female (pink) and male (blue) individuals. The Bengal whipray samples also included fetus samples, which are shown in grey.
